# Supplementary material for: Transcriptional Dynamics Underlying Somatic Embryogenesis in Coffea canephora
Source: Plants (Basel). 2025 Apr 2;14(7):1108. doi: 10.3390/plants14071108 (PMC11991477; doi:10.3390/plants14071108)
Supplement: Supplementary file 1 [file plants-14-01108-s001.zip › Supplementary Material.pdf]

Supplementary Figures

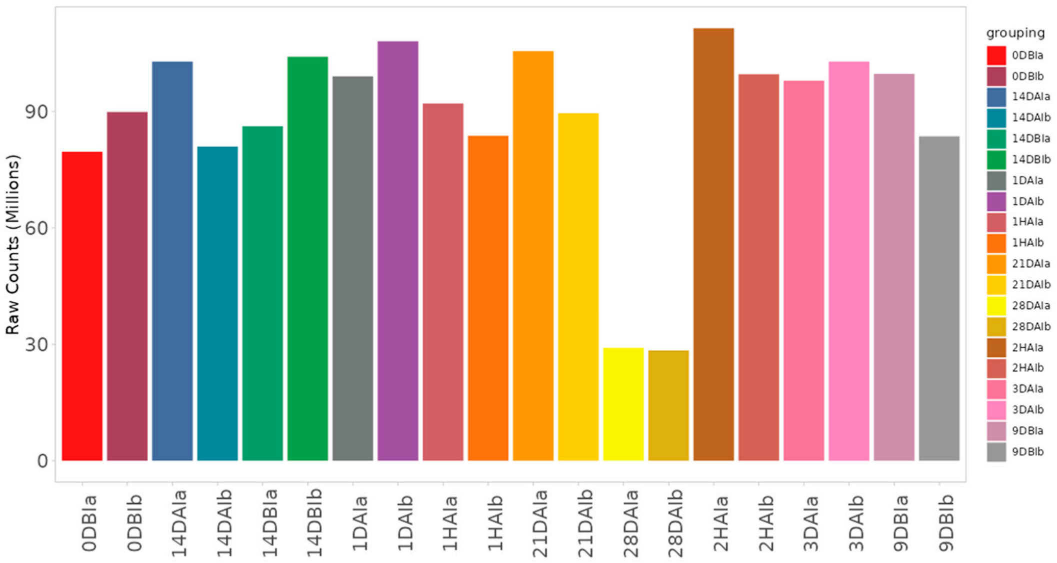

Figure S1. Raw counts for RNA-seq data during somatic embryogenesis in *C. canephora*. This figure provides a visualization of raw RNA-seq count data across various stages of somatic embryogenesis, from 14 days before induction (14DBI) through 28 days after induction (28DAI).

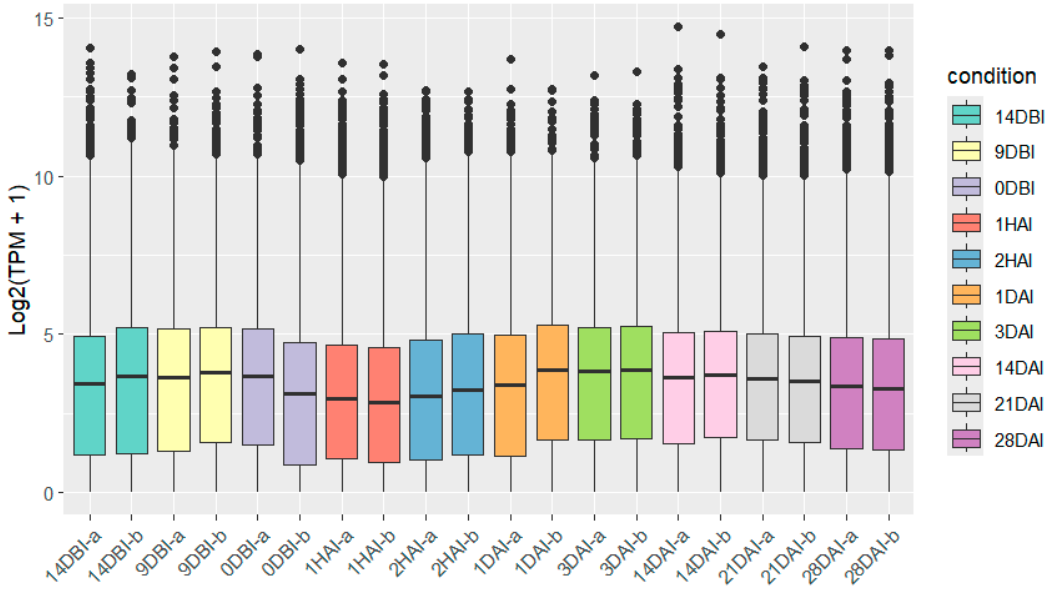

Figure S2. Normalized gene expression counts RNA-seq library from *C. canephora* SE. Boxplot illustrates the distribution of normalized gene expression counts in log2(TPM+1) for each sample of somatic embryogenesis in *C. canephora*. Each box represents the interquartile range (IQR) of expression values for a specific stage, with the horizontal line within each box indicating the median expression level. Each box's upper and lower edges denote the 75th (Q3) and 25th (Q1) percentiles, respectively, while the whiskers extend to the most extreme values within 1.5 times the IQR from the quartiles. Data points beyond this range are plotted as outliers, representing genes with exceptionally high or low expression compared to the entire dataset.

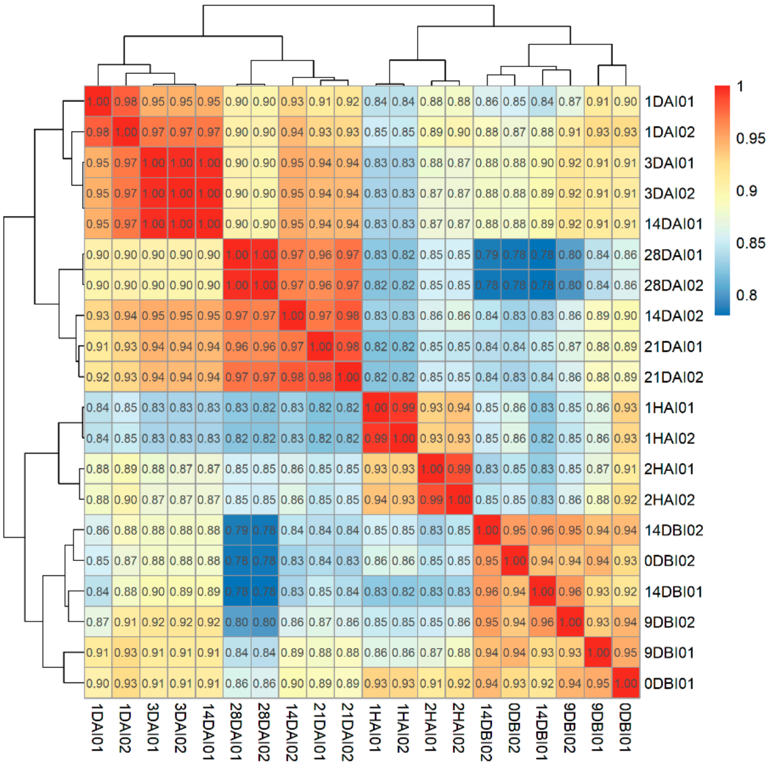

Figure S3. Correlation analysis of VST-transformed RNA-seq expression data. A Pearson correlation matrix displays the pairwise correlation coefficients of VST-transformed raw counts from all samples sequenced.

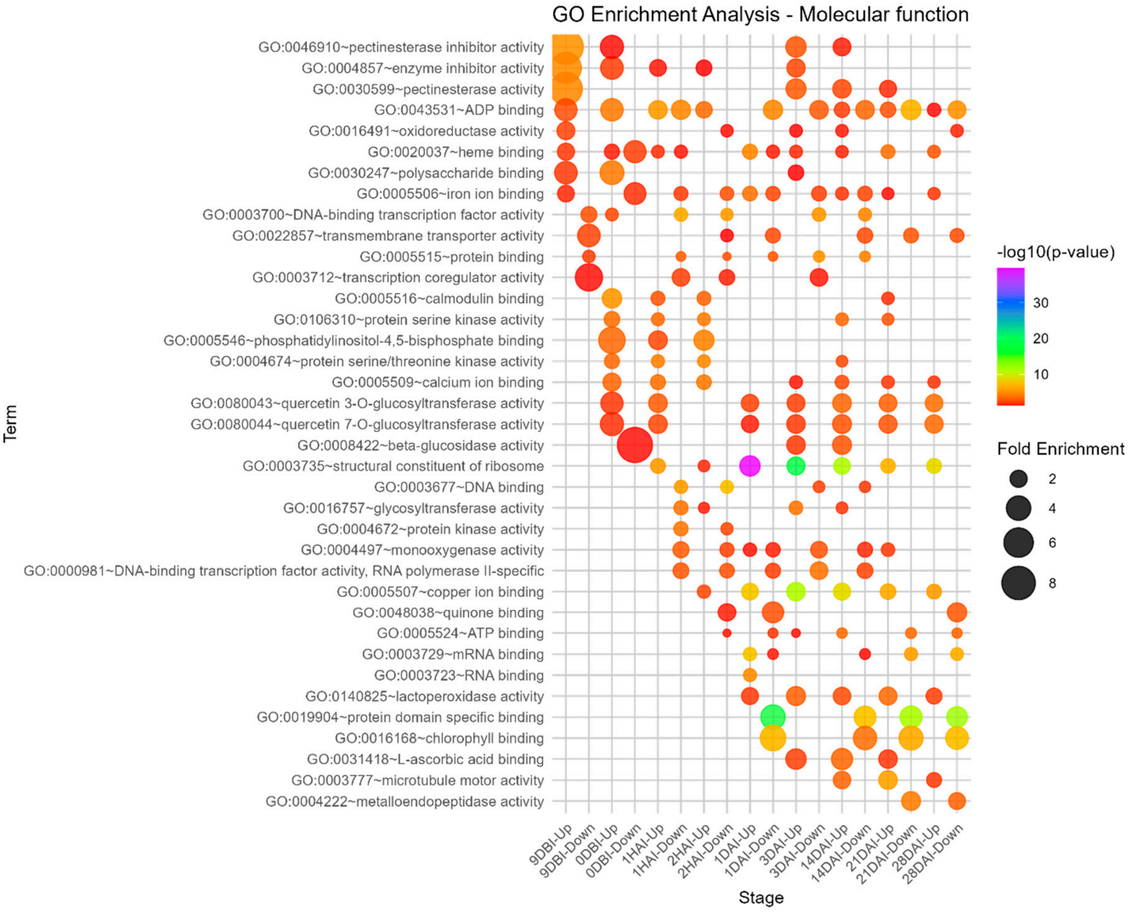

Figure S4. Molecular functions terms enriched from DEGs across different *C. canephora* SE. A dynamic enrichment pattern is observed throughout the process, suggesting stage-specific functional shifts. Enrichment of pectinesterase (PME) and pectinesterase inhibitor (PMEI) activity suggests a potential role in cell wall remodeling induced by the pre-treatment stage. Additionally, the GO:0003712 (transcription regulator activity) term is enriched in downregulated genes, which may correspond to transcription factors (TFs) related to circadian rhythm, light responses, and photosynthesis, similar to the observed within the Biological Process (BP).

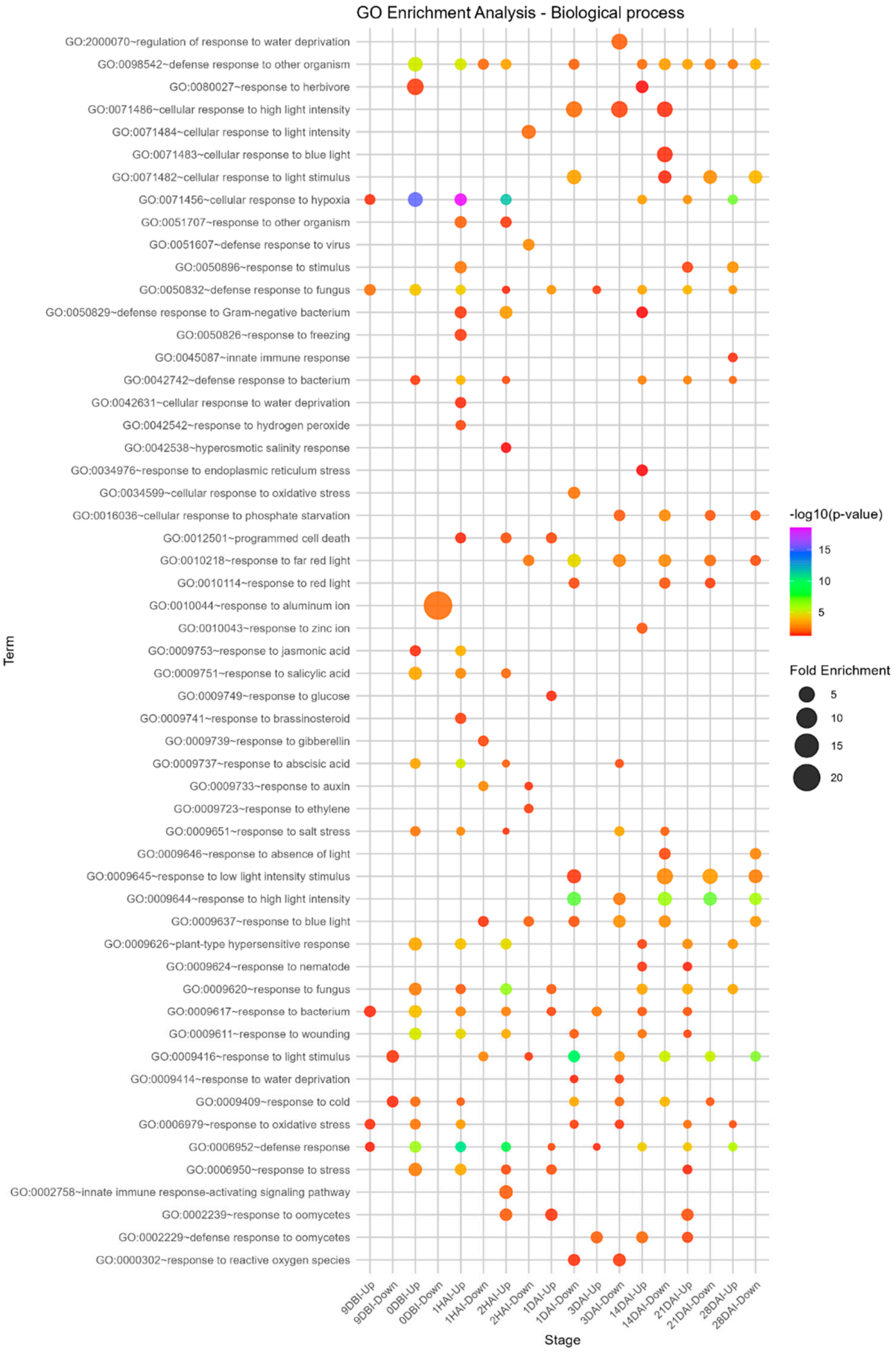

Figure S5. Complete list of Biological Process enriched terms associated with responses to different stimuli. Most GO terms were enriched in upregulated DEGs, some being stage-specific, while others were enriched throughout the process.

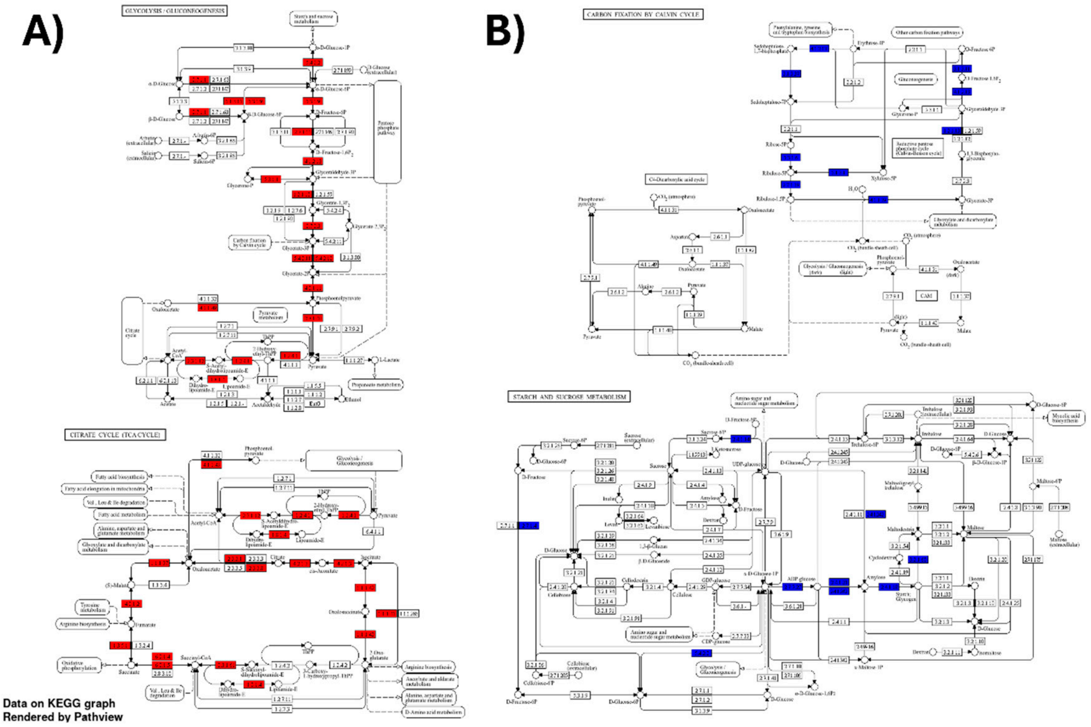

Figure S6. DEGs related to carbon metabolism pathways. Genes involved in glycolysis, a catabolic pathway for energy production, were upregulated. In contrast, genes associated with anabolic sugar metabolism, including photosynthesis and starch biosynthesis, were downregulated. Genes involved in the tricarboxylic acid (TCA) cycle, responsible for generating ATP and reducing power (NADH, FADH2), were also upregulated.

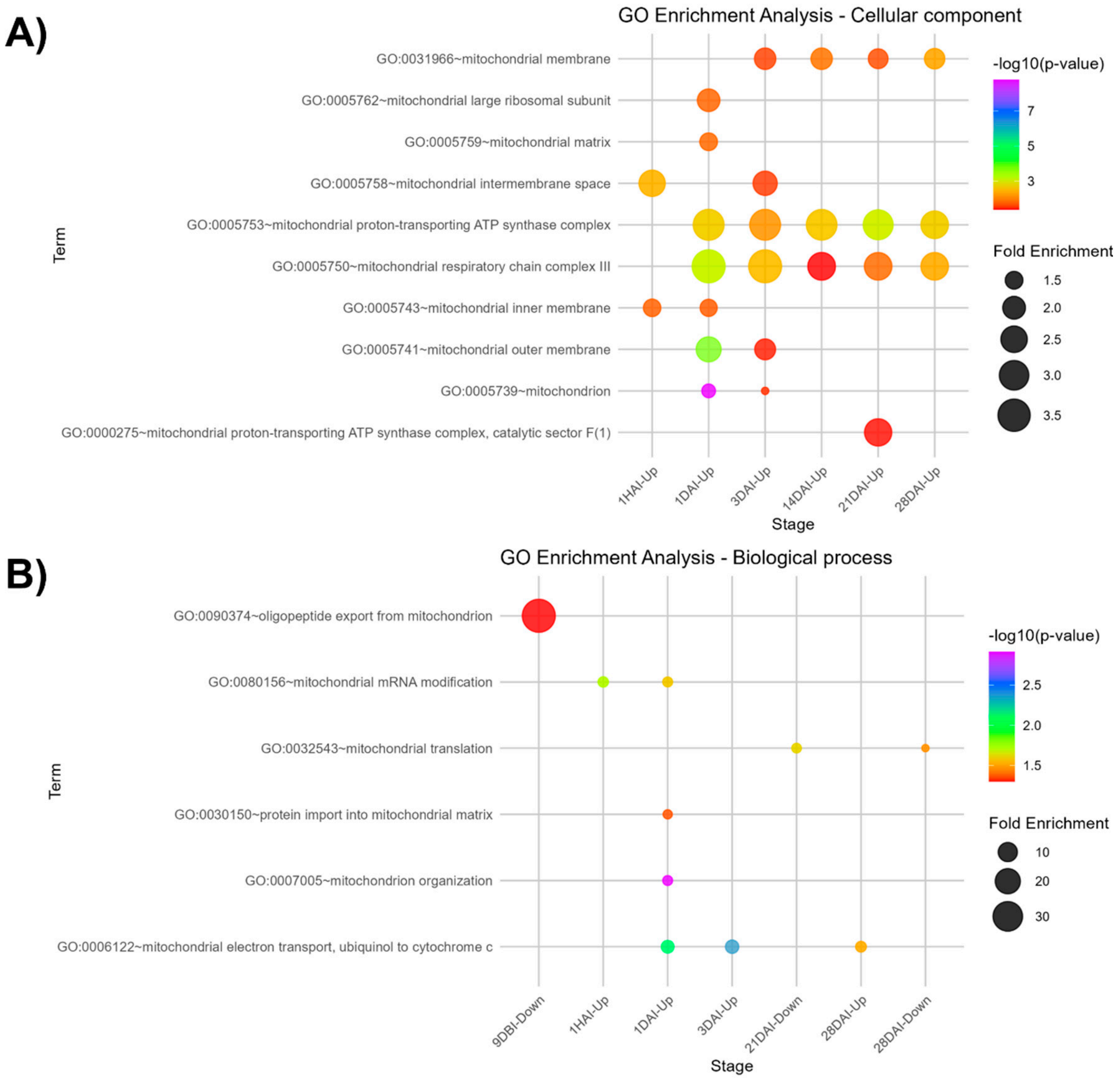

Figure S7. Go enriched terms associated with mitochondria. All mitochondrial-associated GO terms were significantly enriched among the upregulated DEGs.

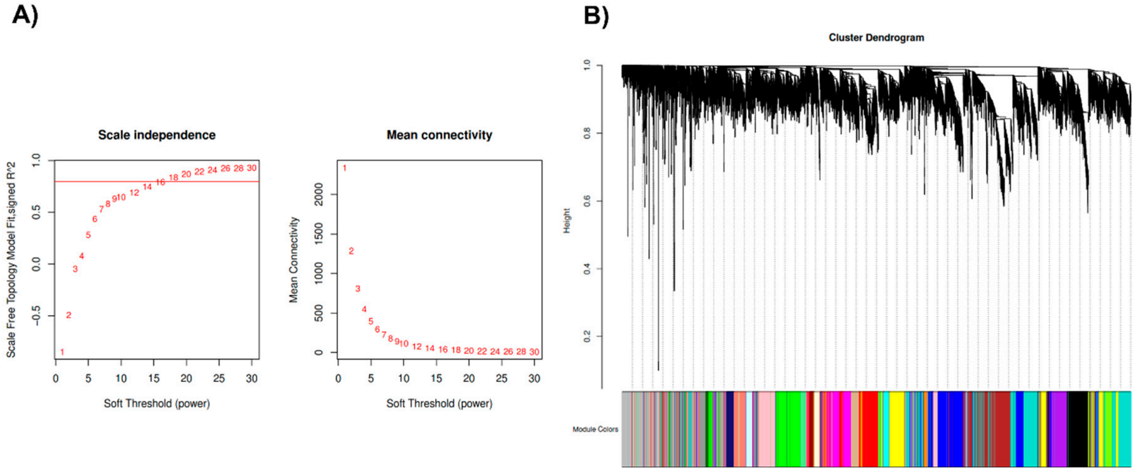

Figure S8. Graphs analyzed during the construction of coexpression networks. A) Soft-threshold power selection for achieving a scale-free topology in the coexpression network. The left panel shows the scale independence as a function of soft-threshold power, with the red line marking the chosen threshold for optimal network topology. The right panel displays the meaning of connectivity, guiding the selection of a power that balances scale independence and connectivity. B) Cluster dendrogram of genes obtained through WGCNA, with modules represented by distinct colors. Each module groups genes with similar expression patterns.

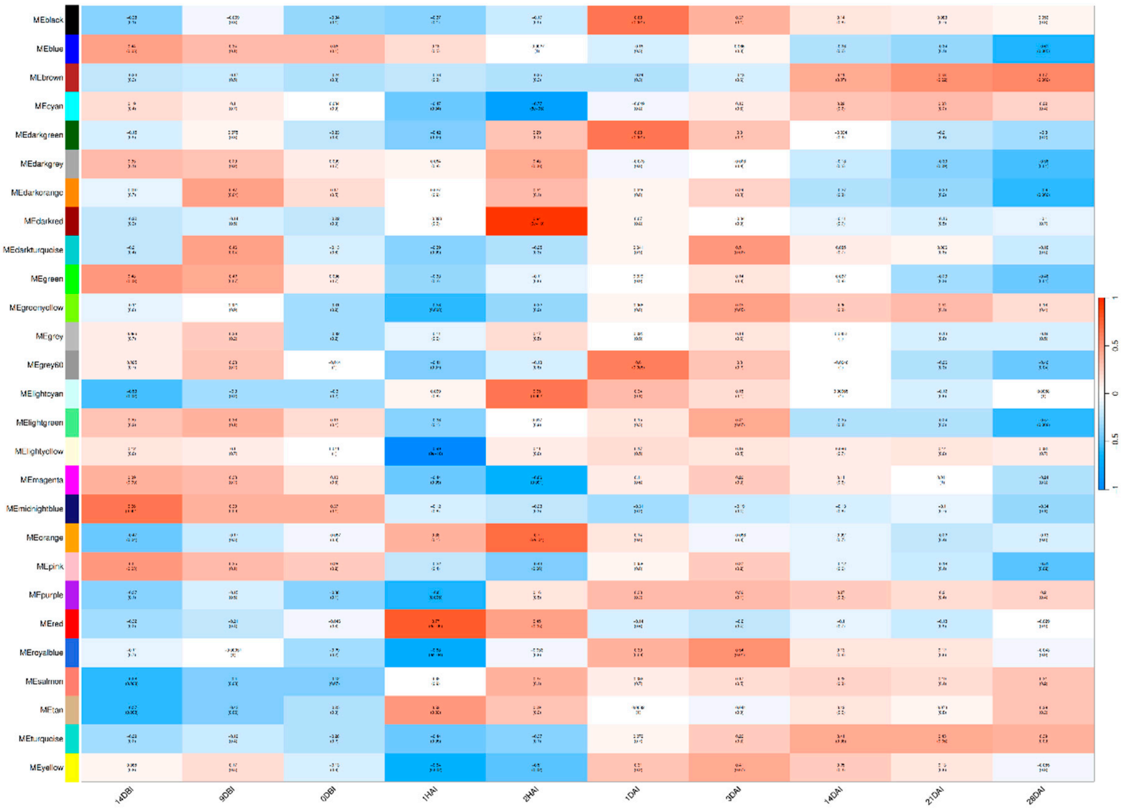

Figure S9. Association of the modules with the sampling points of the SE. This figure displays Pearson correlations between gene coexpression modules and the time-point of the SE process.

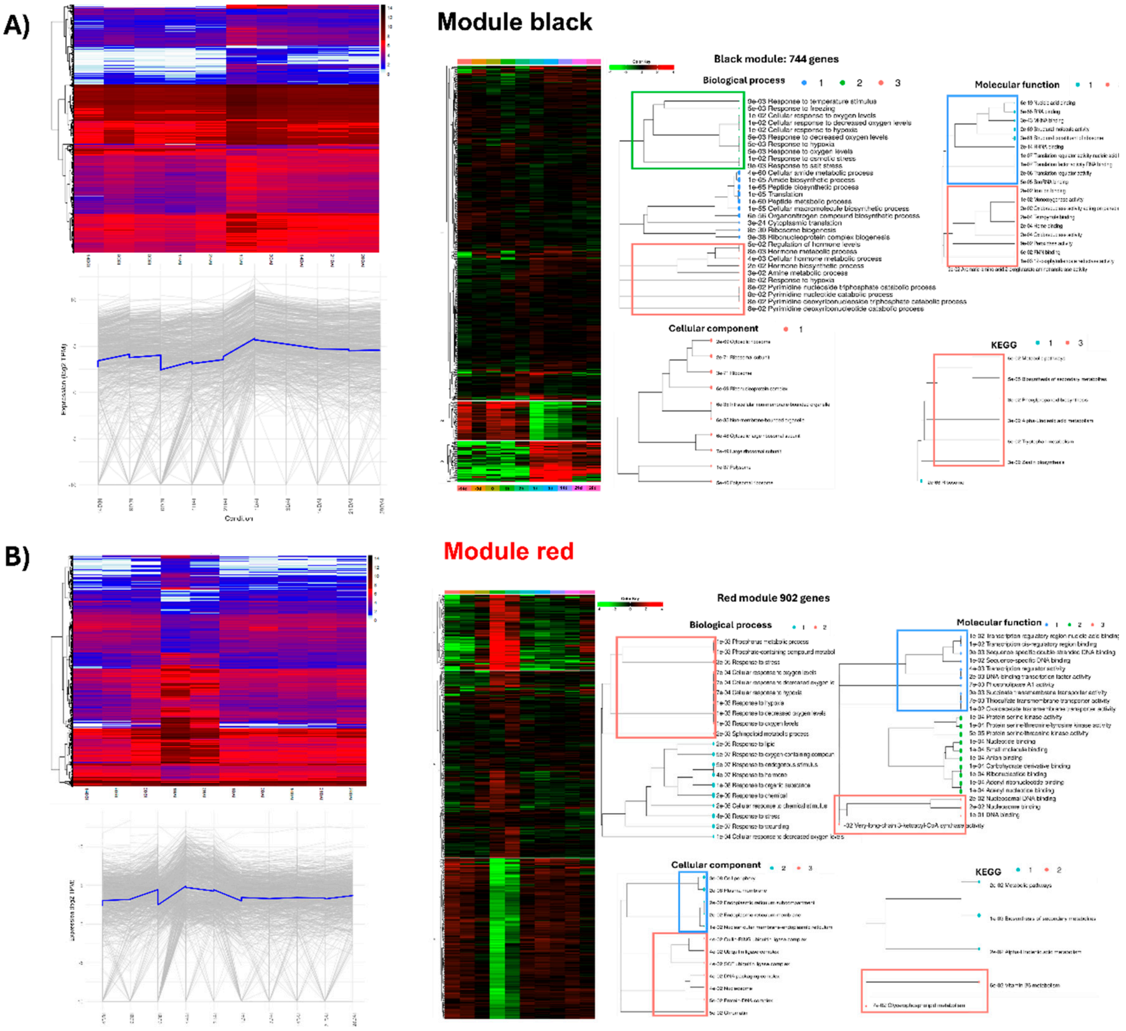

Figure S10. Analysis of black and red modules. A) The black module shows increased expression at 1DAI and 3DAI; k-means clustering identified three. B) The red module displays an upregulated expression at 1HAI and 2HAI; k-means clustering also revealed three groups.

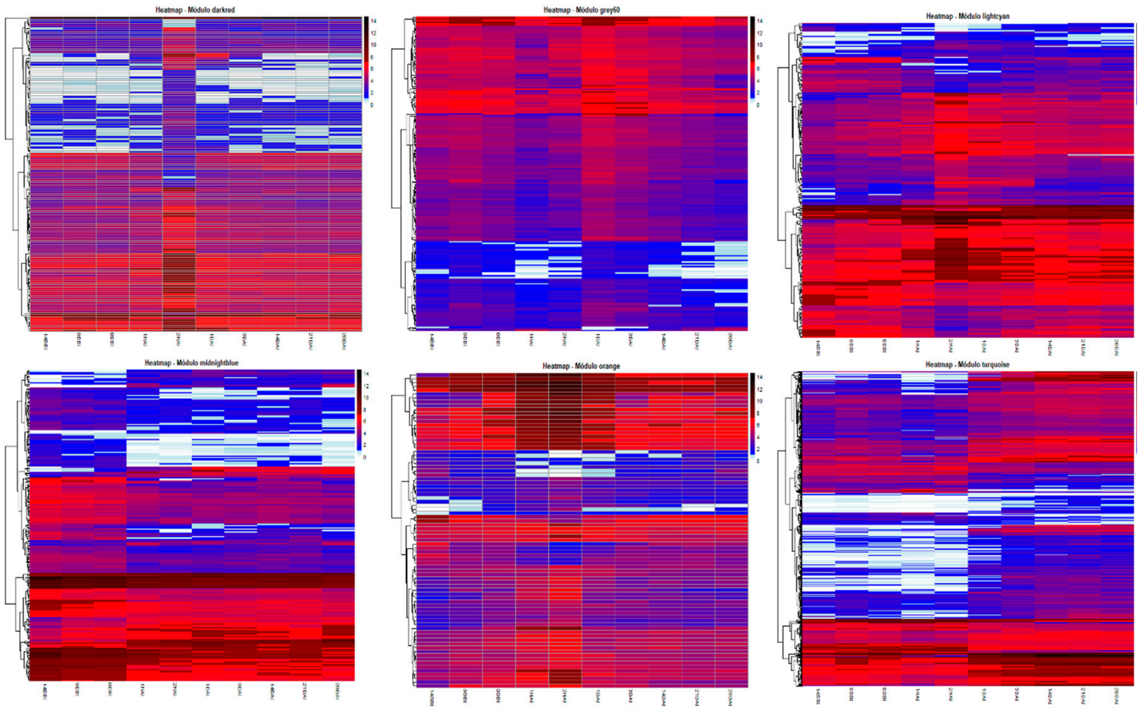

Figure S11. Expression profile of co-expression modules with high correlation (>0.75) to a specific SE stage. A Hierarchical Clustering analysis was performed for each module. Gene expression values are represented in $\log_2(\text{TPM} + 1)$. The color scale transitions from blue to red for expression values ranging from 1 to 8, while values above eight are displayed in a gradient from dark red to black. Genes with an expression value of exactly zero are shown in white, whereas genes with values greater than 0 but lower than 1 follow a white-to-light blue gradient.

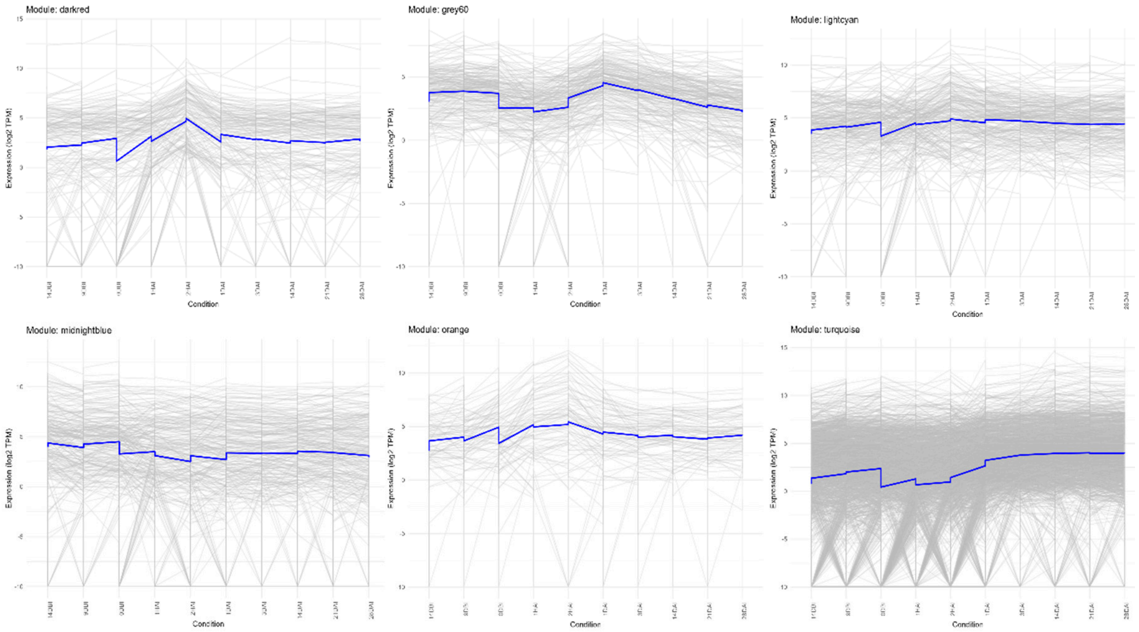

Figure S12. Gene expression dynamics of co-expression modules with high correlation (>0.75) to a specific SE stage. Each panel corresponds to a distinct gene module, where individual gene expression profiles are shown in gray, and the blue line represents the mean expression trend for each module.

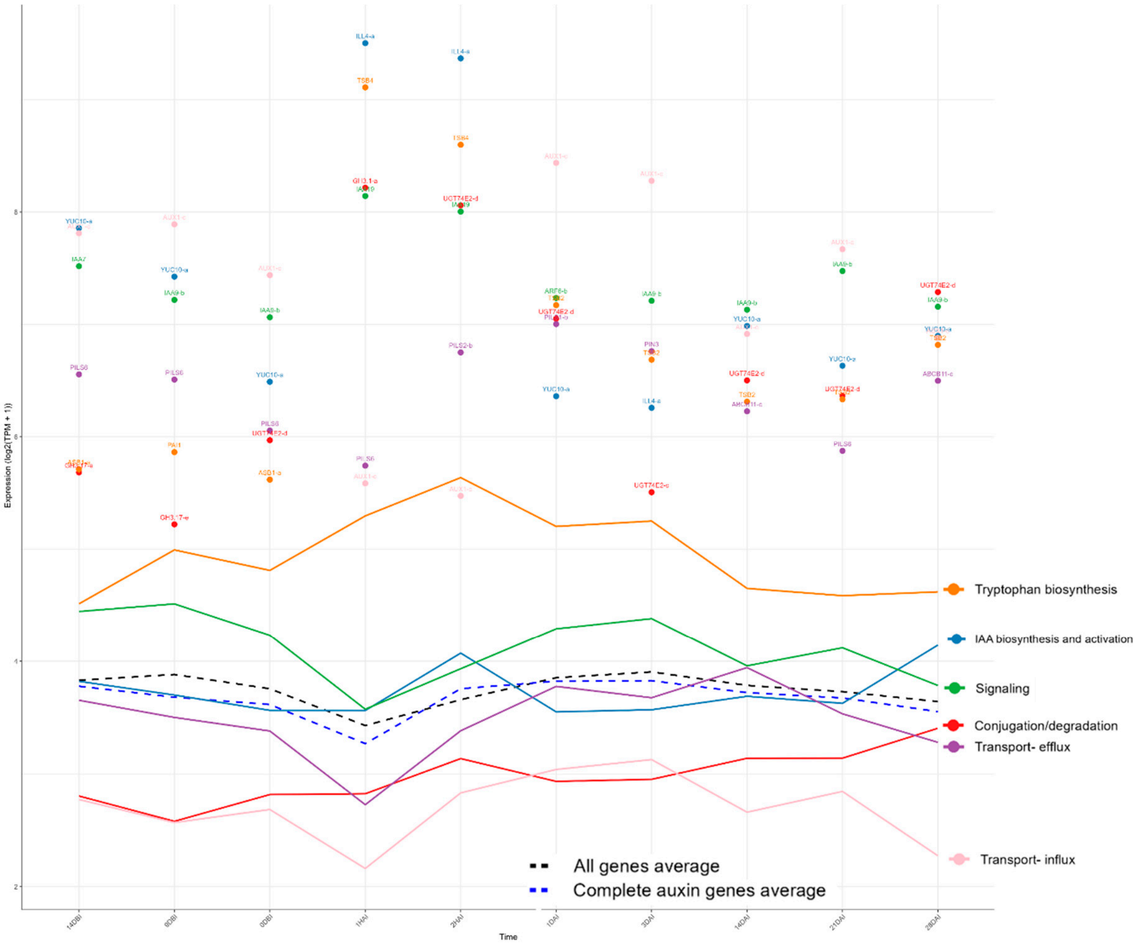

Figure S13. Expression of the average of auxin-related genes from *C. canephora* during SE. Auxin metabolism-related genes were classified into six functional groups. Their average expression ($\log_2$(TPM + 1)) throughout SE is represented as follows: tryptophan biosynthesis (orange), IAA biosynthesis and activation (blue), signaling (green), conjugation/degradation (purple), transport-efflux (red), and transport-influx (pink). The dashed black line represents the average expression of all expressed genes in *C. canephora*, while the dashed blue line represents the average expression of all auxin-related genes.
